# A case of availability bias for COVID‐19 causing scrub typhus diagnostic errors

**DOI:** 10.1002/jgf2.474

**Published:** 2021-06-21

**Authors:** Shiichi Ihara, Kiyoshi Shikino, Masatomi Ikusaka

**Affiliations:** ^1^ School of Medicine Chiba University Chiba Japan; ^2^ Department of General Medicine Chiba University Hospital Chiba Japan

**Keywords:** COVID‐19, diagnostic error, scrub typhus

## Abstract

Although patients with scrub typhus develop a maculopapular rash all over the body, patients with COVID‐19 may also show a similar rash. At the first visit, we did not fully inspect his trunk and extremities under his clothes. Although scrub typhus and COVID‐19 have some similar symptoms, an eschar is a characteristic symptom of the former, and careful inspection is important to distinguish between the diseases.

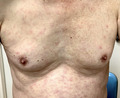

In November 2020, a 70‐year‐old man, living in Chiba, visited our fever outpatient internal medicine clinic with a 7‐day history of fever, cough, headache, arthralgia, and a generalized rash. The patient had dinner with a large number of individuals 10 days before the visit. Physical examination revealed a body temperature of 37.2°C, pulse of 76 beats/min, blood pressure of 102/62 mm Hg, and respiratory rate of 18 breaths/min. Lymph nodes with tenderness were palpable in the bilateral axillae. His breath sounds were clear. A generalized macular eruption with some scattered papules was found from the trunk to the extremities (Figure 1). Laboratory data showed mild elevation of aspartate aminotransferase 81 U/L, alanine aminotransferase 72 U/L, γ‐glutamyl transpeptidase 150 U/L, and C‐reactive protein 13.1 mg/dl.

**FIGURE 1:**
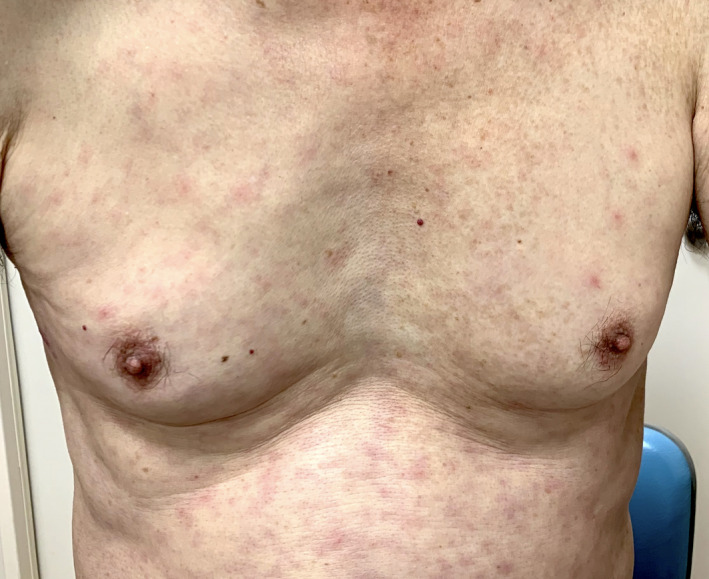
Generalized macular eruption with some scattered papules on his trunk

Under the endemic of SARS‐CoV2, COVID‐19 was suspected based on his persistent fever and cold‐like symptoms, but polymerase chain reaction test for SARS‐CoV2 was negative. The next day, he visited our clinic again because the symptoms persisted. As the patient was engaged in agriculture, we suspected scrub typhus based on the season and his occupation. On careful inspection, an eschar was located on his right lateral chest, which indicated the site of an infected chigger bite (Figure 2). Serological tests showed 1:80 titers of anti‐*Orientia tsutsugamshi* immunoglobulin M and a fourfold increase in titers between paired samples (Gilliam strains). On initiating minocycline administration at 200 mg/day for 14 days, the symptoms resolved completely.

**FIGURE 2:**
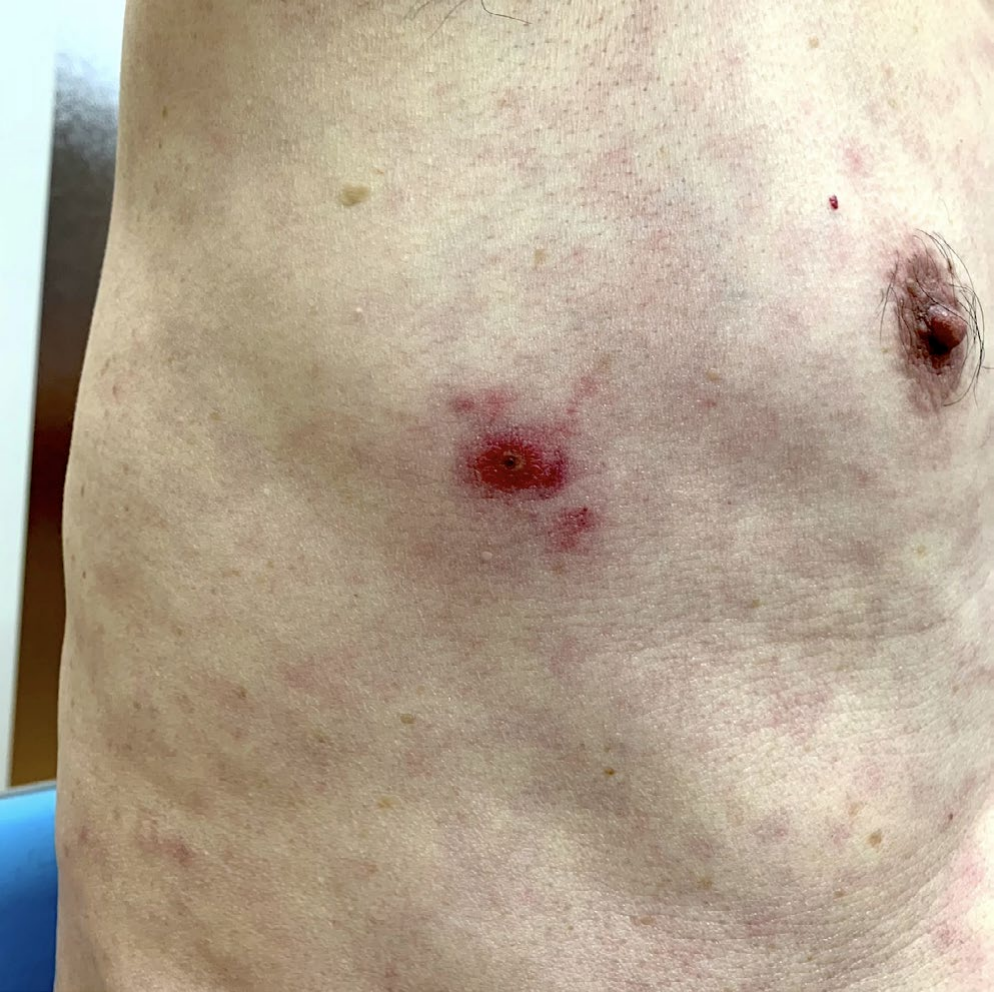
Eschar on his right lateral chest

Scrub typhus is a mite‐borne infectious disease transmitted by *O*. *tsutsugamushi* and is distributed throughout the Asia‐Pacific region.^1^ In Japan, about 400 cases of scrub typhus were reported every year from a broad geographic range.^2^ Besides three major symptoms (fever, an eschar of chigger bite, and skin rash), patients sometimes complain of headache, fatigue, lymph node enlargement, macular or maculopapular rash, and cough (especially in severe case).^3^ However, patients with other infections such as Japanese spotted fever, Epstein‐Barr virus, and COVID‐19 may also show similar symptoms. COVID‐19 also shows many types of skin manifestation such as macular/maculopapular rash, acute urticaria, or vesicular rash.^4^ At the first visit, we did not fully inspect his trunk and extremities under his clothes. Although scrub typhus and COVID‐19 have some similar symptoms, an eschar is a characteristic symptom of the former.^3^

In this case, confirmation of the diagnosis was delayed from the initial visit. As a situational factor of the delayed diagnosis, the physician in the fever outpatient clinic was required to minimize the patient contact time with fully equipped personal protective equipment. The physician was likely to rely on system 1, which is an intuitive clinical reasoning process. In addition, as the number of patients with COVID‐19 was increasing at the time, it is possible that the physician became accustomed to the situation. This could have made it difficult to recall differential diagnoses other than COVID‐19 based on usability heuristics and availability bias.^5^ Even for patients who complain of typical symptoms of COVID‐19, it is important to proceed with differentiation through basic medical history taking and physical examination.

## CONFLICT OF INTEREST

The authors have stated explicitly that there are no conflicts of interest in connection with this article.

## AUTHOR CONTRIBUTIONS

All authors had access to the data and a role in writing the manuscript.

## INFORMED CONSENT

The patient's consent for the case report was obtained after well informed.
